# 3D forensic science: A new field integrating 3D imaging and 3D printing in crime reconstruction

**DOI:** 10.1016/j.fsisyn.2021.100205

**Published:** 2021-10-21

**Authors:** Rachael M. Carew, James French, Ruth M. Morgan

**Affiliations:** a UCL Department of Security and Crime Science, University College London, 35 Tavistock Square, London, WC1H 9EZ, UK; b UCL Centre for the Forensic Sciences, University College London, 35 Tavistock Square, London, WC1H 9EZ, UK

**Keywords:** 3D forensics, Photography, Scanning, Radiography, 3D modelling, Evidence presentation

## Abstract

3D techniques are increasingly being used by forensic scientists in crime reconstruction. The proliferation of 3D techniques, such as 3D imaging and printing being employed across the various stages of the forensic science process, means that the use of 3D should be considered as a distinct field within forensic science. ‘3D Forensic Science’ (‘3DFS’) is therefore presented in this paper as a field that brings together a range of 3D techniques and approaches that have been developed within different areas of forensic science for achieving crime reconstructions and interpreting and presenting evidence. It is argued that by establishing this distinct field, defining its boundaries, and developing expertise, best practice and standards, the contribution of 3DFS to the criminal justice system can be maximised and the accuracy and robustness of crime reconstruction endeavours can be enhanced.

## Highlights

• 3D forensic science (3DFS) is defined as a distinct interdisciplinary field.
• The value of taking a systems level approach to 3DFS.
• 3DFS encompasses 3D tools and approaches, and human decision-making factors.
• Articulating what 3DFS is and what it is for paves a path forward.

## 1 Introduction

As forensic science continues to develop and harness the utility of emerging technologies, the scope and use of three-dimensional (3D) tools at the crime scene and in the analysis, interpretation and presentation of forensic materials is increasing in the criminal justice system [1]. It is therefore important to articulate what this emerging field is, what it is for in the light of what has been achieved and incorporated into practice so far, and what needs to be done going forward to promote the use of best practices to achieve robust 3D forensic science materials, intelligence, and evidence. Therefore, this article sets out a working definition and associated terminology for the emerging field of ‘3D forensic science’ (3DFS); the application of 3D imaging and 3D printing for crime reconstruction purposes. We consider the remit and scope of this field of forensic science, the key actors involved in it, and where it sits within the forensic science process and wider criminal justice system (CJS).

3D reconstructions are currently being used in courts of law [2,3] and are being requested by crime scene investigators to support the presentation of evidence and expert opinion in courtrooms [1]. Alongside the emergence of 3D forensic science, there is a growing need to demonstrate the accuracy and reliability of 3D reconstructions, as well as demonstrating that the techniques used and decisions-made in their production are adequately transparent, reproducible and robust [4]. There is a growing body of published research exploring the use of 3D reconstructions and the methodologies utilised in their creation [[5], [6], [7], [8]]. However, there remain several issues that need to be addressed, such as the terminology used, and the position of 3DFS within the forensic science process, as the position of 3D reconstructions within the forensic science discipline from a conceptual point of view has not yet been fully articulated. In this paper, we consider relevant theoretical frameworks in order to provide a working definition of 3DFS as a forensic science field (what it is) and explore its role within the criminal justice system (what it is for) (akin to the approach outlined by Morgan [9]). In so doing it is hoped that the value of 3DFS and the challenges it faces can be increasingly recognised, and a pathway forward can be found for achieving consistency in future research, the utilisation of 3DFS in crime reconstruction, and the terminology employed in communicating with key stakeholders across the CJS.

## 2 3DFS as a distinct field

Forensic science has historically been a discipline that has developed and evolved in synergy with the needs of its end users and stakeholders. New fields of forensic science have developed as a result, for example, forensic medicine evolved partly in response to needing new methods to answer forensic questions [10], and forensic podiatry has pooled knowledge around feet and footwear to develop a forensically specific evidence base [11]. Forensic fields are also continually evolving as seen by an updated remit for forensic anthropology [12], and the emerging fields of microbial forensics [13], digital forensics [4], and veterinary forensics [14]. These new fields have generally emerged as a need has arisen from casework and the resulting requirement for dedicated, forensically relevant evidence bases to underpin new methods that often draw from other disciplines [15,16]. Interdisciplinary fields such as veterinary forensics utilise knowledge from their parent fields (in this case veterinary science and forensic science) to answer questions of law. This extracted knowledge can take the form of established validated techniques or procedures [15], but they need to be applied in the context of specific forensic evidence bases. Therefore, these new fields of forensically specific endeavours warrant definition as interdisciplinary fields that are distinct from (but intersect with) their parent disciplines.

3DFS draws together methods established in a number of other fields, including forensic radiography, forensic medicine, forensic anthropology, and forensic photography. 3DFS also utilises techniques commonly utilised in these fields such as 3D radiographic techniques, 3D surface scanning methods, photography, and 3D crime scene reconstructions. Considering this interdisciplinary nature of 3DFS, digital forensic science is a useful comparison as an evolving field that also draws upon expertise from various specialisms such as multimedia evidence, voice recognition, facial identification, and image analysis [4]. The generalisable hallmarks of a forensic science field (as articulated specifically for digital forensic science) are using common techniques and/or utilising those techniques to achieve a common goal [4]. Digital forensic science is therefore considered to be a specialisation and field within forensic science. Parallels can be drawn with the field of 3D forensic science, for example, a forensic anthropologist may create a 3D reconstruction from medical imaging data that was generated by a radiographer, and the 3D reconstruction may then be interpreted by a forensic pathologist. Thus, each of these forensic actors are utilising 3D data derived from another discipline, but that data is positioned within a specifically forensic science endeavour to address forensic science questions [15] with a common goal of creating a representation of materials (exhibits, specimens, or evidence) for use in the CJS; this is the field of 3D forensic science.

3DFS brings together methods, knowledge, and expertise in 3D approaches into one cohesive forensic science field. It has emerged (in a similar manner to other fields) as a practitioner-led field, being developed from the ‘ground up’ in response to needs and demands identified in forensic science practice. By articulating what 3DFS is, and what it is for, the approaches drawn from existing knowledge bases of practice and parent disciplines can form forensically relevant evidence bases [15,17] for the application and use of 3DFS in crime reconstructions in a harmonised way in a similar way to digital forensic science. Without a clear articulation of 3DFS as a field, it is possible that opportunities to harness the potential of these tools could be missed as well as there being greater risk of 3D reconstructions being used in the CJS without adequate evidence bases, which could result in misrepresentation of findings, uncertainty around expert evidence, and even unsafe rulings [18].

## 3 Towards a technical definition of 3DFS

As an interdisciplinary science that is concerned with the application of insights to questions of law [15], forensic science brings together many fields including those that consider trace evidence (such as biology and chemistry to address DNA, fingerprints, glass, fibres, etc.), human identification (such as forensic anthropology and forensic medicine), environmental context (such as forensic archaeology and forensic geoscience), human context (such as psychology, sociology, behavioural science), as well as newer fields such as digital and electronic evidence. The forensic science process addresses the transition of materials recovered from a crime scene to their analysis and interpretation and an ultimate presentation to investigators as intelligence or to a court of law as evidence (often known as ‘crime scene to court’) as questions regarding authentication, identification, classification, reconstruction, and evaluation are addressed [4,19]. Given that the forensic science process operates within a wider context that incorporates law, economics, history, culture and policy [17,20] it is important to approach the reconstruction of crime events in a holistic manner that incorporates a consideration of the evidence base upon which each part of the forensic science process is built; the interaction of different forms of materials that contribute to an overarching crime reconstruction; and the role of expertise and human decision-making at every stage and scale [15]. Crime reconstruction thus also captures the role of forensic science as a scientific endeavour [21], that can engage with the complexity of the forensic science ecosystem by including factors from the physical, human, and digital domains, as well as attending to theoretical and practical needs [15].

**Fig. 1:**
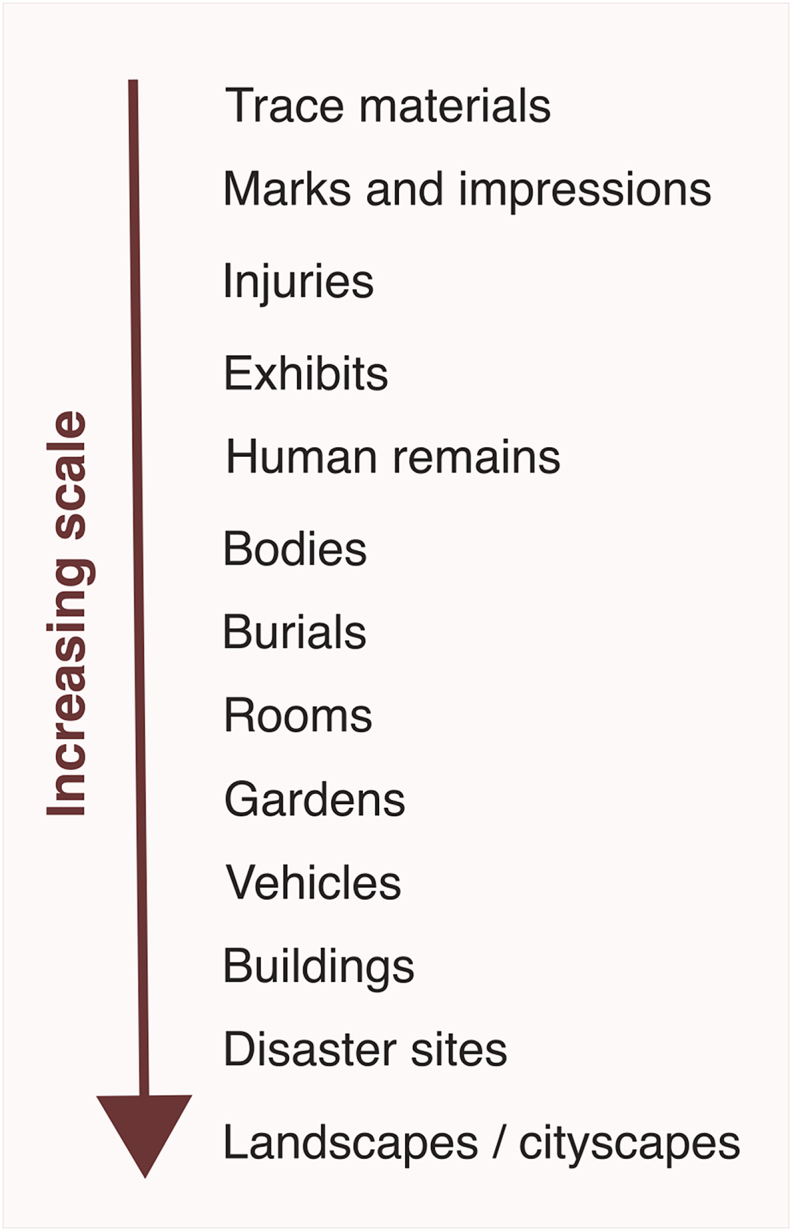
Broad presentation of the increasing scale of forensic materials encountered in 3D forensic science.

Current research addressing 3D imaging and printing in forensic science has used the terms ‘forensic 3D printing’ [22] and ‘forensic imaging’ [23]. However, it is clear that the approaches incorporated by the term ‘3D forensic science’ are broader in scope and application than either of these terms convey when considering crime reconstruction. Therefore, ‘3D forensic science’ (3DFS) brings together the range of approaches involving 3D techniques (such as 3D imaging, 3D modelling, and 3D printing) in crime reconstructions, and includes the many different types of materials being imaged, ranging from marks and impressions, fragmentary human remains, weapons, tools, and bullets (exhibits), vehicles, or entire scenes of crime. It covers different forms of imaging, from surface scanning or clinical imaging modalities [23], as well as the resulting scan or image data, 3D modelling and post-processing stages. 3DFS also incorporates the different types of 3D printing for replicating crime materials [6] and ultimately, the range of 3D presentation methods, including body mapping, animated models, virtual models, and physical replicas. Importantly, 3DFS addresses reconstructions from micro to macro scales (Fig. 1), and the resulting 3D reconstruction can be an accurate representation, or an accurate scaled-up or scaled-down model. 3DFS incorporates a consideration of each stage of the forensic science process from 3D imaging to the presentation of these materials as evidence and incorporates a consideration of the decision-making at each stage of the process that is intrinsic to these approaches, as well as the empirical evidence-bases that underpin research and casework.

## 4 Defining 3DFS

Following the ‘what it is’ and ‘what it is for’ approach from Morgan [9], 3DFS can be considered to be the application of 3D techniques (including 3D imaging and 3D printing) for crime reconstruction purposes, with the goal of producing visual aids for police intelligence or courtroom demonstration purposes. The aim of 3DFS is to complement expert witness testimony and provide an aid that can help the court (judge, juror, and jury) to better understand the evidence being presented. 3DFS may be presented in a courtroom as either a simple visual aid or admitted into substantive evidence where it will carry probative value [24]. Digital or virtual models that appear on a screen or monitor are often termed as ‘3D’, although it is important to note that these are not truly ‘3D’ since they offer only the illusion of depth from stereoscopic vision [5]. Nevertheless, 3D virtual models can be considered as 3D reconstructions since the goal of utilising 3D virtual models is to illustrate materials in a realistic manner with representative reconstructions using visual aids that have (or signify) accurate factors such as depth, colour, movement, or scale.

Field specific definitions are helpful in these interdisciplinary arenas due to overlapping terminologies and technologies. For example, the term ‘reconstruction’ can mean the physical reconstruction of a fragmented object, or the reconstruction of a timeline of events. Within 3DFS, a ‘3D reconstruction’ is the final 3D product that is developed for police intelligence or courtroom presentation purposes. Additionally, the term ‘3D-model’ alone is discouraged due to its ambiguity. Rather we advocate for definitive terms such as ‘virtual 3D model’, ‘physical 3D replica’, or ‘3D printed replica’, whereby the nature of the product (virtual or physical) is implicit.

It is also valuable to define the boundaries of 3DFS in considering which forensic tools are contained within the field, and which are not. For example, body mapping and injury graphics are not often linked with forensic imaging, however, it can be argued that these can be considered part of 3DFS along with 3D imaging and 3D printing, as complementary tools whose purpose is to represent a 3D visualisation for courtroom purposes. The field of digital forensics uses terminology such as ‘digital image forensics’, and the ‘presentation of digital evidence’ that can sound similar to the ‘digital presentation of evidence’, however these are distinct, discrete fields and digital forensics should not be included in 3DFS as its purpose is not to present 3D courtroom materials. Crime scene photography can be included within 3D imaging, particularly with the overlapping utility of photogrammetry (3D reconstructions from photographs) as well new avenues of photogrammetry using drones to capture crime scenes. Traditional crime scene photography is not a part of 3DFS if it is concerned with 2D recording practices. However, the role of crime scene photographers includes many different aspects including, forensic imaging and the generation of virtual 3D models and 3D prints, as well as body mapping and injury graphics. As such, a crime scene photographer may be involved in 3DFS practices some of the time. Extended reality technologies including virtual reality and augmented reality are also beginning to be used in forensic science for analytical and evaluation approaches [25] and police intelligence purposes [26], and considered for courtroom demonstrations [27], and these can be considered within the remit of 3DFS where the reconstructions are being used as a 3D demonstration tool.

There are many steps involved in producing a 3D reconstruction. It is important to acknowledge that each of these steps involves multiple decisions, from choosing an imaging modality, to selecting what data to use, to deciding what appearance to give a final model [5,23,24]. Each of these steps can have important effects on the accuracy and integrity of the final model. Body mapping and injury graphics, in particular, are not always accurate representations of, for example, an injury on a body. Rather, injury graphics can be generated from photographs that are stitched together upon a generic 3D model or anthropomorphic mannequin, or they can be graphical depictions of injuries (i.e., injuries created using digital paint/illustrative tools) presented upon a generic mannequin. Body mapping and injury graphics are frequently used in the CJS in England and Wales, but there is a distinct lack of supporting research or approved methodologies. The importance of having evidence bases to underpin crime reconstructions is well documented [15] and there is a growing forensic evidence base to support 3D imaging and 3D protocols, in particular around the metric accuracy of final models. The final appearance of a 3D reconstruction (such as colour, scale, positional aids, and 3D print material) is vitally important for how it may be interpretated in court when it is presented as evidence [28]. The colour of a 3D reconstruction can range from using a realistic, photo-realistic, or skin-based colours, compared to using a more neutral colour such as white or grey, to a bright eye-catching colour such as pink or blue. Sanitisation (as opposed to realism) is an integral theme to 3DFS and the admissibility of 3D reconstructions in a court of law [3].

Physical 3D reconstructions, such as 3D printed replicas, provide a tangible visual aid that jurors can touch, hold, rotate and potentially use to mimic injuries [29]. A small amount of published research has explored the effects of virtual 3D models and prints on lay people and how this may affect their understanding of expert testimony [30,31]. Case outcomes have been cited as evidence of the efficacy of 3D reconstructions [2,8], however considerably more research is needed to explore these effects, as well as establishing what is the most effective presentation method for each type of crime scene material. For example, currently there is no documented evidence to establish the effect of holding a replica knife or a replica infant skull on a juror. Empirical research from case simulations, systematic analysis of actual jury verdicts, and post-verdict surveys of jurors [32] are all needed to sufficiently evaluate the emotive and/or prejudicial effect(s) as well as the evidential value such tools may have.

## 5 The role(s) and influences of 3DFS actors

In 3DFS, as with other forensic science fields, there are multiple human actors that can be involved in a reconstruction. In 3DFS these can include the individual(s) generating the original imaging data (e.g., forensic radiographer, forensic imaging specialist, forensic photographer); the actor creating the 3D reconstruction (e.g., a forensic pathologist, forensic anthropologist, forensic imaging specialist), printing a 3D replica (e.g., additive manufacturing technician, forensic imaging specialist, engineer), and presenting 3DFS materials in a courtroom. Of the many actors involved, some may be trained to perform certain aspects, others may be specialists in the entire process. A critical component of 3DFS is peer review of the final reconstructions, in a similar manner to other forensic science fields that involve pattern analysis [33] or a significant application of experience and expertise (that incorporates tacit knowledge) such as forensic anthropology. This is particularly important for biomedical data where a forensic pathologist or case-specific specialist such as a paediatric neurologist, may be required to check and confirm the integrity of a reconstruction. The actor performing the peer review may be someone who has already been involved in the reconstruction process, or they could be a new actor bringing a fresh perspective [33].

**Fig. 2:**
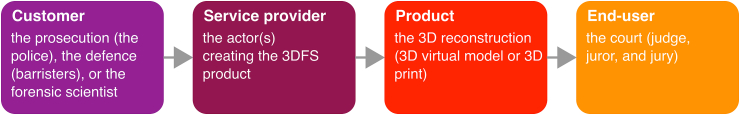
3D Forensic Science (3DFS) process considered through a business lens.

An additional factor when considering the human actors is the consideration of who is supplying the expertise, in comparison to who is making the decisions. Taking a business model lens, the *customer* will be either the prosecution (the police) or the defence (barristers) who acts on behalf of a victim or a suspect. The actor(s) creating 3DFS materials will be providing the *services* and creating a *product* – the final 3D reconstruction. While at the other end of the forensic science process, the court will be the *end-user*; the judge and the jury will be the ones who interact with 3D reconstruction (the product). If a forensic scientist requests or produces a product to assist with their analysis or interpretation of materials, then a forensic scientist could also participate as the customer (and potentially the service provider). Nevertheless, the customer is always distinct from the end-user and as such a more linear pathway can be observed (Fig. 2).

To some extent, each of these actors is supplying their expertise, whether it is the customer in choosing an expert, or the service provider using their expert knowledge to create 3DFS materials, or the end-user using their own judgement to interpret those 3DFS materials. In the strictest sense of ‘forensic expertise’, the service provider is supplying the greatest portion of this and thus holds a lot of influence over the final product. As such, it is possible to consider what is appropriate expertise, what qualifications or certification (if any) are needed. Additionally, it is important to appreciate how a service provider is gaining and developing their expertise from both explicit knowledge gained through formal training, and tacit knowledge gained through experience (perhaps from overlapping fields) [34]. In this way it is possible to identify the nature and source(s) of a particular expertise and recognise how service providers are using evidence bases to support their decisions, and what value or impact that may have on the resulting product [34].

Decision-making is a feature of each stage in a crime reconstruction (Fig. 2). The customer is deciding what product to request, the service provider is deciding what the product may look like, and the end-user is deciding what value the product has. These considerations are valuable because in understanding who is making what decisions at what stage, it is possible to begin to understand the factors that are affecting their decision-making and what influences those factors. For example, identifying the extent to which investigators know what 3DFS can support and how to request the most appropriate analysis or product for the crime scene material in question becomes key to the deployment of 3DFS during investigations. In a similar vein, the type of guidance that is available to assist decision-makers in commissioning or producing the most reliable, accurate 3D reconstruction will impact the value of 3DFS in the justice system.

Multiple service providers currently exist in 3D Forensic Science, partly a result of the new and interdisciplinary nature of the field. However, this is also a result of inconsistencies within the current procurement and attainment of 3DFS services. As a result of the fragmented forensic science ecosystem in the UK, where procurement and provision of services is provided by both in-house (within the police) and external providers [9], there has been a combination of existing forensic specialists (such as crime scene photographers) being trained in-house to produce 3DFS materials, in addition to, external forensic or academic experts providing independent specialist services and products (when they may have limited awareness of the forensic context). Having a range of types of expertise may not necessarily present an issue in courts of law but given the heterogeneity of expertise that can exist it is clearly important that the remit and source of expertise is declared to the court.

In establishing 3DFS as a distinct field, it may be possible to improve the clarity of establishing who has the relevant expertise to produce and present 3DFS in courtrooms. Experts can then be specially trained and/or certified in 3DFS, to present robust reconstructions that support the CJS. Acknowledging the field of 3DFS will also help to address issues such as relying on expert ‘opinion’ alone to support presentations of 3DFS materials in courtrooms. For example, a forensic specialist may currently present a 3D reconstruction without providing any supporting evidence as to why that 3D reconstruction is accurate. In contrast, the same expert could present 3DFS materials supported by empirical evidence bases that demonstrates how that model was generated and why it is accurate and reliable in the forensic context. Having a unified field opens up opportunities for 3DFS accreditation, certification, and standardisation schemes. Currently, there are no such schemes in the UK to cover 3DFS explicitly. This represents a key challenge for forensic science in terms of staying up to date with and harnessing the value of emerging technologies and fields [35]. In addition, a recognised field creates opportunities for dedicated 3DFS publications, journals or special issues that consolidate and bring together research outputs which can contribute to developing an evidence base for the field.

## 6 3DFS in a holistic forensic science process

**Fig. 3:**
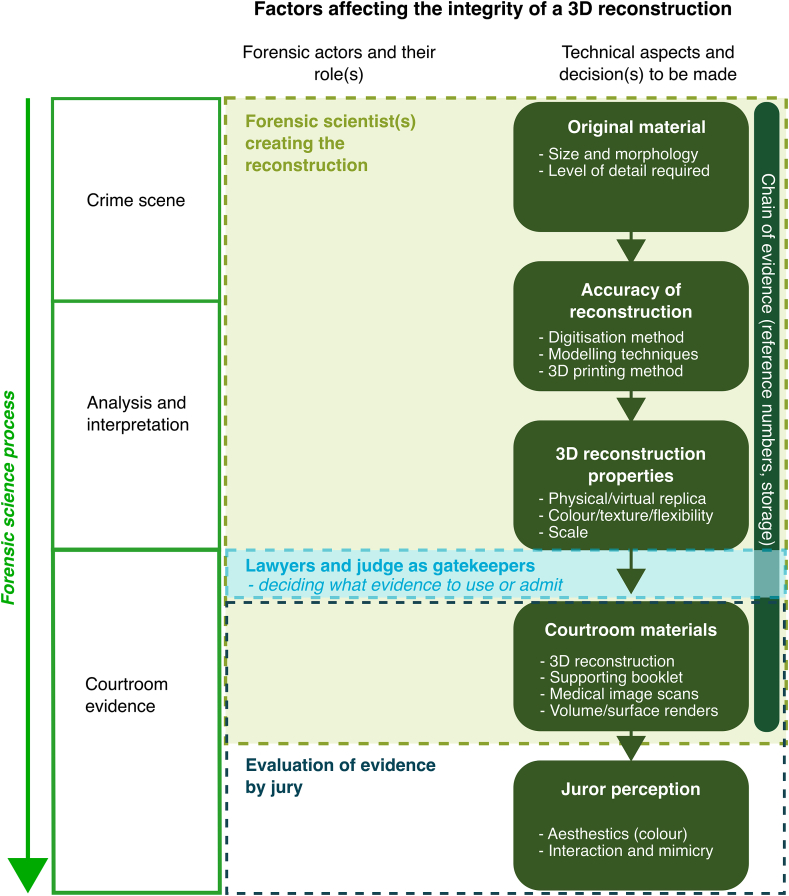
An illustration of the factors affecting the integrity of a 3D reconstruction from crime scene to courtroom.

The evaluation of the roles and influences of 3DFS actors are integral to the ‘analysis and interpretation’ portions of the forensic science process [15]. The use of 3D reconstructions has been largely reserved for the presentation of courtroom evidence (the final stage of the forensic science process) [1]. Although it is acknowledged that certain 3D reconstructions are used more often in police intelligence, for example in developing 3D maps for operations planning. The use of 3D reconstructions in the analysis or interpretation of materials by a forensic expert has been fairly limited in practice. Interpretations from 3D skeletal reconstructions are not always supported by evidence bases and experts are advised to use analysis from the original materials or from more established techniques, such as medical image data, microscopy, or histology [37]. Consequently, the use of supporting ‘courtroom materials’ is included in Fig. 3. While 3DFS is providing useful 3D tools, these are not yet fully developed in all areas.

In considering 3DFS at each stage of the forensic science process, it is possible to identify the different pertinent factors involved at each stage and how these may affect the integrity of a final 3D reconstruction (Fig. 3). For example, if a 3D reconstruction exhibit is created by ‘forensic scientist(s)’ they may be any 3D forensic science expert that is engaging with materials from the crime scene to court (or indeed separate actors throughout that process). The ‘forensic scientist(s)’ has a significant impact on many technical aspects of the 3D reconstruction from crime scene through to the courtroom. The judge acts as the gatekeeper in deciding what evidence can be admitted and lawyers will make decisions about what visual aids to use, and once those materials have been presented it is often juries who are tasked with evaluating them (along with everything else presented during the hearing) to reach a verdict. Each decision made will impact subsequent parts of the process and its outcomes [36], so taking a holistic view is valuable as it offers the opportunity to identify issues within the production system and to deliver transparency throughout that process.

## 7 Conclusion

It is important to consider 3DFS as a distinct field of expertise and special interest. 3DFS is an interdisciplinary field of forensic science that encompasses the application of 3D techniques for crime reconstruction purposes and provides tools for representing sanitised 3D visualisations for courtroom purposes to help the court to better engage with and understand evidence.

Looking forward, established frameworks to support the new field of 3DFS will be needed. This work has already started with increased collaboration between research, policy and practice [1], the drive to create more empirical evidence-bases to support 3DFS approaches and outputs at every stage of the forensic science process [24], and guidance being developed to address the use and limitations of each 3D technique [29]. It is clear that the value of 3DFS is growing, and it is well placed to become an established field of forensic science that can be used reliably in crime reconstruction.

## CRediT authorship contribution statement

**Rachael M. Carew:** Conceptualization, Writing – original draft, Writing – review & editing. **James French:** Conceptualization, Writing – review & editing. **Ruth M. Morgan:** Conceptualization, Writing – review & editing.

## Declaration of competing interest

The authors declare that they have no known competing financial interests or personal relationships that could have appeared to influence the work reported in this paper.
